# The Academic, Societal and Animal Welfare Benefits of Open Science for Animal Science

**DOI:** 10.3389/fvets.2022.810989

**Published:** 2022-04-12

**Authors:** Christian Nawroth, E. Tobias Krause

**Affiliations:** ^1^Institute of Behavioural Physiology, Research Institute for Farm Animal Biology, Dummerstorf, Germany; ^2^Institute of Animal Welfare and Animal Husbandry, Friedrich-Loeffler-Institute, Celle, Germany

**Keywords:** 3R, Open Access, preprints, pre-registration, Registered Report

## Abstract

Animal science researchers have the obligation to reduce, refine, and replace the usage of animals in research (3R principles). Adherence to these principles can be improved by transparently publishing research findings, data and protocols. Open Science (OS) can help to increase the transparency of many parts of the research process, and its implementation should thus be considered by animal science researchers as a valuable opportunity that can contribute to the adherence to these 3R-principles. With this article, we want to encourage animal science researchers to implement a diverse set of OS practices, such as Open Access publishing, preprinting, and the pre-registration of test protocols, in their workflows.

## Introduction

Open Science is a widely applied practice in certain scientific fields such as psychology or ecology (1). It makes research from an early stage on accessible to other researchers, is key to increase collaborations, and can help to avoid potential biases in the publication system. In addition, a significant proportion of research is directly or indirectly supported through public funding. It is therefore also imperative to make obtained scientific findings freely accessible, and the way this knowledge is generated as transparently as possible to increase trust in the scientific system and its outcomes. However, the traditional scientific publication system, as being still the most influential in many scientific fields, involves a number of obstacles likely to hinder the process of freely available scientific knowledge and transparency by including delays and restricted access in the proliferation of protocols and results, such as pay-walled articles, long reviewing times, and publication biases toward novel positive findings, (2, 3). While many tools are available to improve the transparency and accessibility of the scientific process and the subsequent research outcomes, the most powerful tool available is likely the implementation of Open Science practices (4). Open Science covers various aspects of the scholarly process, ranging from e.g., Open Access publishing of research articles, to providing Open Data and Protocols, to Open Science Evaluation (open peer review) and Open Science tools such as Open Source software–with the goal of building on, reusing and openly discussing scientific knowledge.

The application of these Open Science practices has been assessed and is growing rapidly in fields such as psychology and ecology (1), while their current state and progress in other fields, such as animal science is, to our knowledge, not systematically assessed. While general academic and societal benefits of Open Science might be apparent (and more or less generalizable across disciplines), we here will further argue that the implementation of Open Science practices will also benefit the field of animal science by a stronger adherence to the 3R principles to reduce the number of animals in research, refine protocols and methods, and replace animal studies with animal-free alternatives (5).

## Why is Open Science Important for Animal Scientists?

Research in animal science often involves animal experiments that require the housing and testing of animals that can, to a considerable degree, compromise their welfare. As such, these experiments are also under special scrutiny and observation of the legislation and the public. Accessible and transparent research, e.g., *via* Open Science, offers options to decrease/mitigate some of these conflicts between welfare, animal experimentation and public awareness. Beside academic and societal points, we here aim to outline animal welfare as another important reason for a better implementation of Open Science practices in animal science.

From an academic point, Open Science can enable researchers to improve the scientific communication and dissemination of test protocols, data, and publications that, in turn, can lead to increased efficiency and thus save costs in research, for example *via* faster sharing of novel, and identification of outdated, research protocols. Also, from a societal point, knowledge (and the way knowledge is generated) that supports the need for improvements in animal husbandry and management, and measures the success of different interventions in achieving animal welfare goals, needs to be accessible and transparent to policymakers, farmers, media and other stakeholder to change and further improve legislation and animal protection laws. Open and transparent reporting of research findings, accompanied by adapted public engagement strategies, can furthermore help to maintain public trust for science in general. Such public engagement strategies should be used to actively communicate research to the public to faster spread new insights and potentially also trigger broader discussions on certain topics. Here, open and transparent reporting of research, by researchers, can be perceived as more trustworthy.

**Figure 1:**
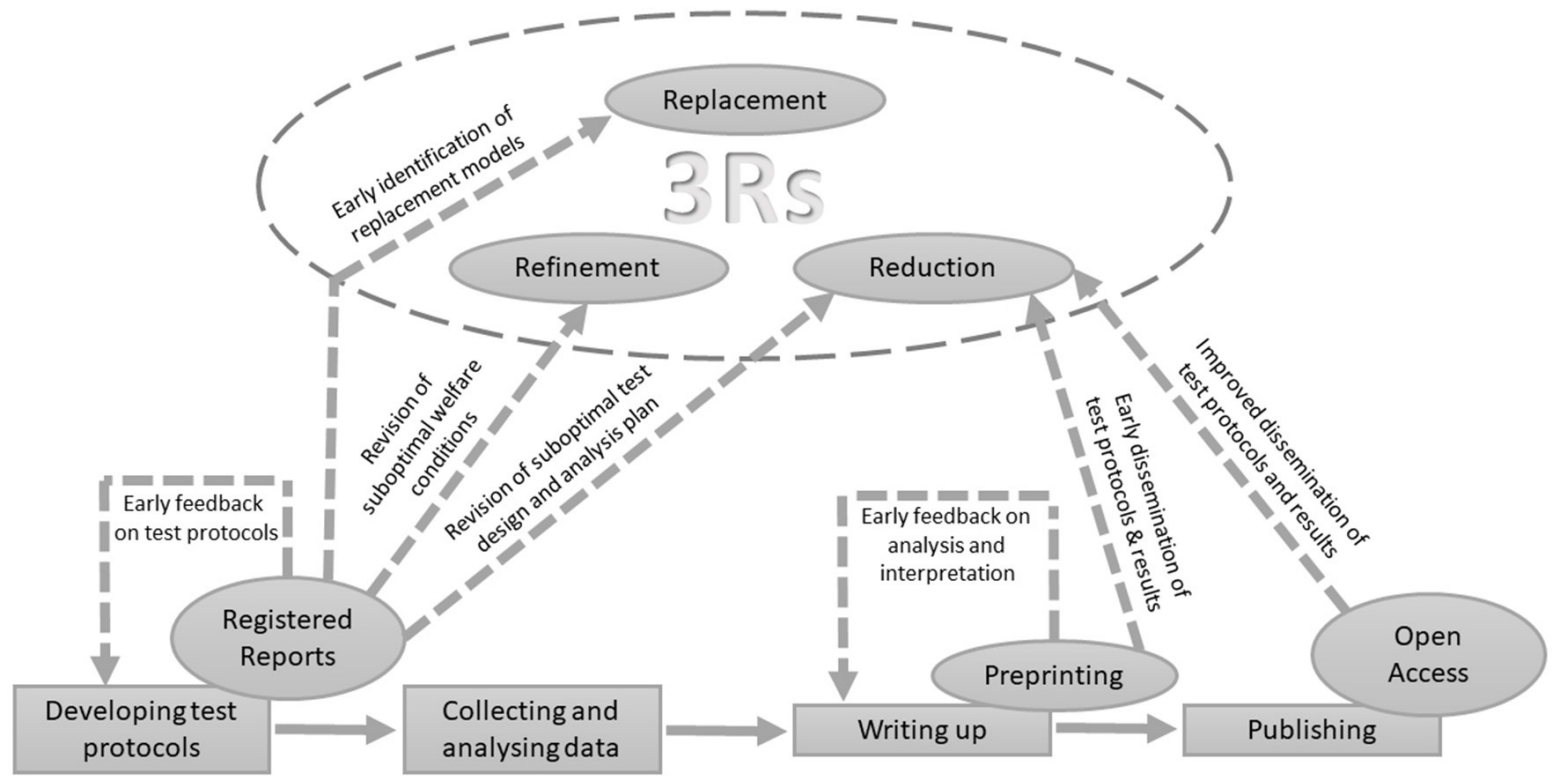
Graphical illustration of the most apparent animal welfare benefits by implementing Open Science practices such as pre-registrations/Registered Reports, preprinting, and Open Access publishing.

A point that is specifically important for animal science researchers is that Open Science can also offer benefits for the welfare of the animals that are used in research and respective animal experiments. The increased transparency of reporting research protocols and findings could lead to a significant reduction in the number of individual animals tested in animal science, thus complying with the 3R principles [Refinement, Reduction and Replacement of animals used in experimental research (6)]. This is especially relevant for research that exposes animals to welfare conditions that are compromised by the study design. These Open Science benefits on animal welfare might initially focus most on the “reduce” part of the 3R principles. In the long-term, however, “refinement” and “reduction” could also be addressed as e.g., early review, as well faster dissemination, offer the potential to improve the implementation of valid replacement models, as well as improved husbandry and management designs (see Figure 1 for an overview).

We here will outline in detail three main Open Science practices: i.) pre-registrations, ii.) preprints and iii.) Open Access publishing, and briefly summarize their academic and societal benefits, but most importantly focus on their potential benefits for animal welfare.

### Pre-registrations and Registered Reports

Questionable research practices such as p-hacking (i.e., changing statistical methods until a *p*-value below the, often default, significance threshold of 0.05 appears) and HARKing (Hypothesizing After Results are Known) are highly problematic (7) as they can mislead researchers and the public that a biological relevant effect exists where there isn't any, and can thus undermine scientific integrity of the scientific community. With pre-registrations of studies, and its corresponding protocols and analysis plans, authors can deposit their hypotheses and study designs on pre-registration servers before they start data collection (8). These pre-registrations can also be submitted to a journal and undergo peer-review with the resulting article type being called “Registered Reports”. Comparisons between traditional, non-pre-registered articles and these Registered Reports show that effect sizes of the latter are far below the former–showing that particular biases, such as publication bias, have caused inflation in published effects in traditional, non-pre-registered articles (9, 10).

This early documentation of the hypothesis, and the test and analysis protocol, can not only increase the credibility of results and their interpretation, but also increase subsequent publication speed (*via* Registered Reports, RR). Academics can already receive a provisional acceptance by a journal based on their submitted Registered Report, streamlining the submission and publication process. Early review at this stage can thus reduce the time to make scientific knowledge available as it reduces the amount of time from completion to publication, e.g., as rejections from journals at the final stage of RRs are the exception. At first glance, this process shifts the time for peer-review only to another stage of the publication process. However, at this early pre-data collection stage, additional synergistic effects are possible, i.e., as detailed study plans are also required for the ethical approval process and/or grant proposals. More robust, peer-reviewed study plans are likely to have also higher changes to be granted/get approval. It should be noted that these pre-registrations have the option to only be made public after a certain embargo period, so that the research idea will remain protected until the publication of the results. Pre-registration, and Registered Reports in particular, can also ensure that negative results from animal experiments become available to the public. For society, pre-registrations, and the peer feedback that they can receive when submitted as a Registered Report, can save monetary resources because potential pitfalls in the study design might be identified before data collection begins, and thus can be corrected. Most importantly, however, is that pre-registrations can increase the confidence and trust in scientific findings by the public due to a prior reliable documentation of the test protocol and analysis plan before starting data collection and analysis.

However, can pre-registration RRs also contribute to the 3R principles, and thus to animal welfare? We here propose several points why this might be the case. Feedback on RRs allows identification of redundant study designs before data collection starts and thus having a direct impact on the number of animals used in research (11). Early feedback on the study design can also flag outdated animal models, raising the chances to find potential replacement models to answer the same study question (see Figure 1). Preregistrations can potentially also simplify the work of ethics committees as committee members could use the peer-review recommendations from the Registered Report regarding the scientific rigor and robustness of the study design. Pre-registrations could thus be potentially linked to ethics approval processes, streamlining the process of scientific assessment and ethical considerations using experimental animals for a proposed experiment. Depending on national guidelines regarding ethical oversight of animal testing, barriers such as specific bureaucratic hurdles and the language used in these reports need to be assessed in order to find common ground.

Although the number of journals offering Registered Reports is increasing, not all fields are affected equally by this increase (12); e.g., to our knowledge, no journal dedicated to animal science is currently offering this article type (see Table 1).

**Table 1:** Current Open Science practices in animal science journals (compiled 01/2022)–a brief overview: we selected an exemplary and non-representative sample of Hybrid Open Access journals that represent different publishers and cover animal science while not specialized to a certain animal group.

**Journal**	**Publisher**	**Embargo for post-prints (accepted doc version) according to https://v2.sherpa.ac.uk/romeo/**	**Preprinting possible (according to guidelines for authors and https://v2.sherpa.ac.uk/romeo/)**	**Offers Registered Reports (according to guidelines for authors)**
Animal Science	Cambridge University Press	immediately for author's homepage 12 months for repository	Yes	No
Animal Welfare	Universities Federation for Animal Welfare (UFAW)	12 months	Yes	No
Applied Animal Behaviour Science	Elsevier	immediately for author's homepage 12 months for repository	Yes	No
Journal of Applied Animal Welfare Science	Taylor and Francis Online	immediately for author's homepage 12 months for repository	Yes	No
Journal of Animal Breeding and Genetics	Wiley	12 months	Yes	No
Journal of Animal Science	Oxford University Press	immediately for author's homepage 12 months for repository	Yes	No

*We assessed whether Open Access approaches such as pre- and post-printing are supported by the journals and whether they offer the opportunity to submit Registered Reports*.

### Preprints

Peer-review is one of the most important steps for quality control of the scientific literature and is supposed to serve as a key gatekeeper for high-quality and rigorous science. As crucial as peer-reviewing is at the journal-level, it is often very time consuming and can significantly delay the publication of new research findings. The peer-review system is often at its limits and editors often struggle to find suitable expert reviewers in a timely manner. Peer-review is also sometimes tilted to favor more famous names in the field (13). Furthermore, if a manuscript is repeatedly rejected by different outlets (e.g., for reasons that are not linked to its scientific merits, including studies that support the null hypothesis), this can lead to additional delays and stretch the peer-review system to its edges. Ultimately, this can in some cases lead the authors to the decision to not publish their manuscripts at all. During these delays, protocols and data are usually not available and thus hamper the proliferation of this information not only to the scientific community, but also stakeholders in society (such as policy makers and consumer). To make their manuscript available before peer-review, researchers can submit a preprint to a preprint server. Preprints are research manuscripts that are shared openly before or at submission to a journal, i.e., prior to peer-review. Preprint archives usually assign a digital object identifier (DOI) or/and a permanent URL (Uniform Resource Locator), making them also citable for other researchers.

For academics, the benefits of this preprinting process include the rapid dissemination of academic work, which can facilitate open access to the literature for a wider audience. It also allows fast feedback from peers, which may than be addressed prior to formal peer-reviewing, and thus can facilitate scientific collaborations (14). As feedback can vary depending on research field and specific topic of the preprint, a wide dissemination is recommended in order to increase feedback from peers. In terms of scholarly impact, manuscripts submitted as preprints tend to receive later on, when published by a journal, a higher citation count (15). The number of downloads of a preprint also predicts the journal impact factor of the subsequently published manuscript (16). Some might argue that preprinting will encourage research theft of the initial idea (and/or data) and others might publish it before the original authors (i.e., “scooping”). While this option can ultimately not be excluded, it is extremely unlikely as preprint archives provide a publicly available DOI and/or URL timestamp to a manuscript. In turn: by posting a citable preprint, a researcher can stake a claim to the work they have done.

For society, preprints offer also benefits: early insights into cutting-edge topics can be picked up (with the given caution that they have not been peer-reviewed yet) by interested lay people or the media, as recently seen in COVID-19-preprints (17). However, clear communication and badges are required that these manuscripts are not yet peer-reviewed and have to be taken with the respective caution. Preprints thus offer an effective way to share novel findings, especially if they affect urgent responses to problems that stakeholders are facing.

Beside the potential benefits for academia and society, preprints also offer benefits for animal welfare. They have the potential to lead to a decrease in the number of animals used in research by indicating early which protocols have been used (and are likely to be more successful to answer specific questions) and which labs have already done research on a topic of interest. This can likely reduce unintended duplication of research methods that are not suitable to answer a specific question at hand (see Figure 1). Preprints could, in the long-term, also lead to an earlier and wider dissemination of novel practices to refine husbandry and to the early identification of valid replacement models.

Right now, preprinting is allowed by most publishers and journals in the field of animal science. However, with some policies it is often not clear whether they target self-archiving after acceptance of a publication, or actual preprinting before peer review [(18) see also Table 1].

### Open Access

Open Access refers to the removal of major obstacles, such as pay-walls or subscription fees, to accessing, sharing, and re-using any output of research. Open Access of papers can be achieved *via* different routes. The so-called Gold route refers to journals that publish all their articles under an Open Access license, effectively making them freely accessible at the point of publication. This route is accompanied by article processing charges by the journal; thus, the authors need to pay and not the reader. However, this route risks that research findings from groups with little internal funding and/or from developing nations have lower chances to get published this way. However, many journals offer waivers that can help to level the playing field and some funding agencies support universities to proliferate Open Access. The so-called Green route refers to author self-archiving, in which post-prints of peer-reviewed articles or non-peer-reviewed preprints are posted online to an (institutional and/or subject) repository or to a personal website; there are no financial costs for authors nor for readers *via* the Green route. This Green route is often dependent on journal or publisher policies on self-archiving (see Table 1). Some journals allow instant self-archiving of the post-print of a now published, but pay-walled article, on a researcher's own or institutional webpage, effectively increasing proliferation of these findings. Nonetheless, many publishers require an embargo period before a post-print can be deposited in public repositories (see Table 1).

Many of the established journals in animal science are, however, adhering to the traditional pay-walled publishing model, with the option to publish with so-called Hybrid Open Access licensing. However, only a relatively small proportion of articles in these journals is published with such Hybrid Open Access licensing, probably because many institutes pay for Open Access only in fully open journals as otherwise institutions have to pay subscription fees and additional article processing fees. All definitions of Open Access type (Gold, Green, Hybrid, Diamond, Bronze, Closed) are described in Piwowar et al. (19).

Open Access, from an academic perspective, implies that research is available at any time to any researcher worldwide, regardless of the economic situation of their institute (20). It also allows researchers to legally share their own work to a wider audience. Furthermore, it seems to pay-off for authors to publish their research with an Open Access license, as studies show that these items are viewed more often and are cited more often compared to pay-walled articles (21). An issue to be solved in the future are the costs to authors for publishing under a Gold Open Access license; many institutes or universities have specific budgets or contracts for these journals and some funding agencies also provide specific funding for such publishing. However, it is not free to any researcher yet to have such funding available.

The societal impact of Open Access is high, in particular for advocating research to policy makers and advancing citizen science initiatives (22). Everyone interested can have access to the full paper. For example, farmers, agricultural companies and instructors who aim to transfer the most recent research findings into farm husbandry and management practices can benefit from this freely available research. However, Open Access publishing alone might not be sufficient to reach all stakeholders, so an active public engagement strategy is key to speed-up the process of transferring scientific findings to the society.

From an animal welfare point, openly available peer-reviewed research papers, protocols, and research data have the potential to reduce the amount of new animal experiments conducted by avoiding redundant designs due to a faster and wider dissemination of the findings (see Figure 1). Easier access to the peer-reviewed literature might also ease meta-conclusions with a potentially smaller number of studies and maybe more heterogeneous results. A wider and more open dissemination might also ease up identifying novel enrichment items or management practices (refinement), but also valid replacement models.

## Summary

The benefits of Open Science for general academic and societal issues are relatively generalizable across scientific disciplines. Open Science practices here have the potential to increase public trust in research findings due to heightened transparency. In addition, animal science, as all research involving experiments with animals, has the obligation to reduce, refine, and replace the usage of animals in research, the so-called 3R-principles. An efficient adherence to these principles would be improved by transparently publishing research findings, data and protocols. This can be accomplished *via* the means of a variety of Open Science practices. Open Science should thus be considered by animal science researchers as a valuable opportunity that can contribute to the adherence to these 3R principles. We stress the need for future investigations on the crossroads of Open Science and the specific issues that experiments in the field of animal science often face, such as frequent adaptation of novel test protocols. We also want to encourage animal science researchers to implement a diverse set of Open Science practices in their workflows, a notion that could be, next to other measures, implemented by providing workshops and courses in research training for Early Career Researchers (i.e., graduate students and early postdocs), but also for more senior PIs in the field.

We acknowledge that implementing Open Science practices can be accompanied with specific downsides, such a potentially increased time investment (followed by a steep learning curve) and an initial lack of conformity by peers and colleagues on this issue. Nevertheless, engaging in these practices will move the field, and us as researchers, forward compared to the current situation of not being (fully) open and transparent about animal research [see here for steps how to implement Open Science in animal science, (23)].

## Data Availability Statement

The original contributions presented in the study are included in the article/supplementary material, further inquiries can be directed to the corresponding authors.

## Author Contributions

CN and ETK: conceptualized and wrote and edited the manuscript. Both authors contributed to the article and approved the submitted version.

## Funding

CN was supported by a grant from the Deutsche Forschungsgemeinschaft (DFG, LA 1187/6-1).

## Conflict of Interest

The authors declare that the research was conducted in the absence of any commercial or financial relationships that could be construed as a potential conflict of interest.

## Publisher's Note

All claims expressed in this article are solely those of the authors and do not necessarily represent those of their affiliated organizations, or those of the publisher, the editors and the reviewers. Any product that may be evaluated in this article, or claim that may be made by its manufacturer, is not guaranteed or endorsed by the publisher.
